# Determining the Influencing Factors on Acceptance of eHealth Pain Management Interventions Among Patients With Chronic Pain Using the Unified Theory of Acceptance and Use of Technology: Cross-sectional Study

**DOI:** 10.2196/37682

**Published:** 2022-08-17

**Authors:** Paula Stoppok, Martin Teufel, Lisa Jahre, Caroline Rometsch, Diana Müßgens, Ulrike Bingel, Eva-Maria Skoda, Alexander Bäuerle

**Affiliations:** 1 Clinic for Psychosomatic Medicine and Psychotherapy LVR-University Hospital Essen University of Duisburg-Essen Essen Germany; 2 Center for Translational Neuro- and Behavioral Sciences University of Duisburg-Essen Essen Germany; 3 Department of Experimental and Clinical Medicine University of Florence Florence Italy; 4 Department of Neurology Center for Translational Neuro- and Behavioral Sciences University Hospital Essen Essen Germany

**Keywords:** eHealth, eHealth interventions, Unified Theory of Acceptance and Use of Technology, UTAUT, chronic pain, pain management, acceptance

## Abstract

**Background:**

Chronic pain is a complex disease with high prevalence rates, and many individuals who are affected do not receive adequate treatment. As a complement to conventional therapies, eHealth interventions could provide many benefits to a multimodal treatment approach for patients with chronic pain, whereby future use is associated with the acceptance of these interventions.

**Objective:**

This study aims to assess the acceptance of eHealth pain management interventions among patients with chronic pain and identify the influencing factors on acceptance. A further objective of the study is to evaluate the viability of the Unified Theory of Acceptance and Use of Technology (UTAUT) model and compare it with its extended version in terms of explained variance of acceptance.

**Methods:**

We performed a cross-sectional web-based study. In total, 307 participants with chronic pain, as defined according to the International Association for the Study of Pain criteria, were recruited through flyers, posters, and web-based inquiries between December 2020 and July 2021. In addition to sociodemographic and medical data, the assessment included validated psychometric instruments and an extended version of the well-established UTAUT model. For statistical analyses, group comparisons and multiple hierarchical regression analyses were performed.

**Results:**

The acceptance of eHealth pain management interventions among patients with chronic pain was overall moderate to high (mean 3.67, SD 0.89). There was significant difference in acceptance among age groups (*W*=9674.0; *r*=0.156; *P*=.04). *Effort expectancy* (β=.37; *P*<.001), *performance expectancy* (β=.33; *P*<.001), and *social influence* (β=.34; *P*<.001) proved to be the most important predictors of acceptance. The extended UTAUT (including the original UTAUT factors as well as sociodemographic, medical, and eHealth-related factors) model explained 66.4% of the variance in acceptance, thus supporting the viability of the model. Compared with the original UTAUT model (*performance expectancy*, *effort expectancy*, and *social influence*), the extended model explained significantly more variance (*F*_25,278_=1.74; *P*=.02).

**Conclusions:**

Given the association between acceptance and future use, the knowledge of the influencing factors on acceptance should be used in the development and promotion of eHealth pain management interventions. Overall, the acceptance of eHealth pain management interventions was moderate to high. In total, 8 predictors proved to be significant predictors of acceptance. The UTAUT model is a valuable instrument for determining acceptance as well as the factors that influence acceptance of eHealth pain management interventions among patients with chronic pain. The extended UTAUT model provided the greatest predictive value for acceptance.

## Introduction

### Background

According to the International Association for the Study of Pain, pain is defined as “an unpleasant sensory and emotional experience associated with, or resembling that associated with, actual or potential tissue damage” [1]. Pain is considered chronic when it persists or recurs for >3 months [2], whereby different time periods can be found in the literature. Keeping the definition in mind, chronic pain is not only a diagnosis in its own right but also occurs as a symptom in connection with numerous somatic and mental diseases. This in turn explains the high prevalence of chronic pain worldwide [3,4], with the number of diseases with chronic pain continuing to increase because of changing demographics [5,6].

Chronic pain negatively affects quality of life, is associated with sleep disorders and mental illness, and leads to increased mortality [4,7,8]. Chronic pain produces extraordinary costs stemming from absence from work, loss of productivity, hospital stays, physician consultations, diagnostics, and treatment [9,10]. Although chronic pain affects a large part of the population worldwide, the care provided for patients seems to be insufficient. The period between the appearance of the first symptoms of a chronic pain condition and the start of pain therapy averages 4 years in Germany [11]. Patients who have to wait for >6 months for treatment show a worsening of their quality of life and a higher risk of being diagnosed with comorbid depression [12]. It is important to come up with new solutions for adequate health care for patients with chronic pain to improve the imbalance between the increasing numbers of such patients and the need for treatment, which remains unmet because of the comparatively small number of licensed pain therapists [11].

In clinical medicine, eHealth approaches can offer a variety of different treatment options. eHealth is a broad term that includes the use of electronic options such as mobile phones and computers to expand medical care [13]. Although eHealth interventions offer many benefits in the management of patients with chronic pain, they also present difficulties. First, not all patients have the same technical requirements. Thus, the use of eHealth is dependent on the socioeconomic status of patients [14]. In addition, difficulties could arise because of the possible physical distance between physician and patient or a lack of trust in the technology and concerns about data security [15-18]. eHealth interventions can provide a cost-effective extension of chronic pain health care that is flexible in terms of time and location [19]. Another benefit is the reduced requirement for caregiver resources in comparison with conventional face-to-face treatment. The anonymity provided by eHealth might be able to lower the threshold for seeking therapy and reduce stigmatization [20]. Especially during the COVID-19 pandemic, care for patients with chronic pain became severely limited with a significant decrease in face-to-face treatment options because of pandemic restrictions [21].

Across different patient groups, research has shown that eHealth interventions have outcomes that are comparable to those of face-to-face therapy [22-24], in particular in the treatment of chronic pain [24]. Especially eHealth interventions that focus on internet-delivered cognitive behavioral therapy [25] and internet-based psychoeducational and therapeutic programs have provided satisfactory improvements in various patient-relevant outcomes such as pain magnitude, disability, and comorbid depression [26].

The initial results of such interventions have been promising [27,28], and it is certain that the efficacy of, as well as adherence to, new eHealth interventions strongly depend on patients’ intention and perseverance to use them [29].

Numerous offerings, including pain apps, are already available, and a review comparing pain apps between 2011 and 2016 shows an increase in the number of apps [30,31]; yet, among the large number of apps, just 8% were developed with the assistance of health care professionals, and almost none of the reviewed apps have been thoroughly tested for effectiveness with regard to pain-related health outcomes. None of the apps offered self-management options [32]. The quality of the offerings is therefore questionable, and need-oriented care cannot be guaranteed in the absence of evidence.

Considering that three-fourths of users discontinue using an app within 48 hours of downloading it [33], it is important for clinical practice to identify the factors that drive patients to use, and stay adherent to, web-based interventions such as mobile apps. For the successful use of eHealth interventions, it is important to measure the acceptance of potential users because acceptance represents the key predictor of actual use. Acceptance in this case can be understood as intention to use and can therefore be operationalized as behavioral intention (BI) [34]. One way to increase acceptance is to involve the target group in the development process of eHealth interventions [35].

Until now, knowledge on the acceptance of eHealth interventions has been inconsistent. Before the development of the Unified Theory of Acceptance and Use of Technology (UTAUT), there was no validated instrument to determine the influencing factors of acceptance [16,36]. The UTAUT now provides a validated and well-established instrument that can be used to identify predictors that influence the acceptance of eHealth interventions. Barriers to acceptance are also identified; therefore, they can be avoided or adequately addressed in the development of eHealth interventions [34,36,37].

The UTAUT was developed as a combination of different models to estimate the intention to use technology as well as predict actual use behavior. It is suitable for assessing the likelihood of eHealth use in different groups [16,37,38]. The instrument consists of four core predictors as direct determinants of user acceptance and use behavior: performance expectancy (PE), effort expectancy (EE), social influence (SI), and facilitating conditions (FCs), where FCs influence actual use but not the intention to use, also called BI (acceptance); PE covers the extent to which a patient believes that they will benefit from using eHealth offerings; and EE represents the expected effort associated with the use of eHealth offerings. The factor SI describes the degree to which patients believe that persons of trust think they should use eHealth offerings. FCs define the patients’ perception that an organizational and technical infrastructure exists to enable the use of eHealth offerings. The first three predictors PE, EE, and SI are direct determinants of acceptance; that is, the intention to use pain management apps. FCs are direct determinants of actual use behavior. To the best of our knowledge, the UTAUT has never been used among patients with chronic pain.

### Objectives

To further develop urgently needed eHealth approaches in pain care, the lack of knowledge about the acceptance of eHealth pain management interventions must be remedied. Therefore, this study aimed to assess the acceptance of eHealth pain management interventions among patients with chronic pain and identify the factors that influence their acceptance of such interventions. The acceptance is influenced by sociodemographic factors such as age [16,38,39], education [16,39], and employment [38], as well as prior eHealth use [39] and depressive symptoms [16,38,39]. An additional objective of the study was to examine the viability of the UTAUT model with its three predictors of acceptance (PE, EE, and SI) and compare it with the extended UTAUT model used in this study. The hypotheses of the study are as follows:

1. Previous studies have indicated overall low [16,40] to moderate [38,41,42] acceptance of eHealth interventions in different target groups. We expected similar results regarding acceptance of eHealth pain management interventions in patients affected by chronic pain.
2. We hypothesized a positive relationship between the UTAUT model’s three core predictors of acceptance (PE, EE, and SI) and acceptance, as demonstrated in previous research [34,38,42]. Therefore, we expected the results to confirm the viability of the UTAUT model.
3. Age [16,38,39], gender [39], education [16,39], occupational status [38], prior eHealth use [39], internet anxiety [41], and mental health variables [16,38,39] are considered to be influencing factors on acceptance. We expected divergent levels of acceptance in the different subgroups in accordance with sociodemographic and health-related factors.
4. Furthermore, we anticipated a significantly higher level of explained variance using the extended UTAUT model compared with the original UTAUT [42].

## Methods

### Study Design and Participants

A cross-sectional study was conducted to determine the predictors that influence the acceptance of pain management apps. Between December 2020 and July 2021, participants were recruited with flyers and posters distributed at hospitals and practices of physicians and physiotherapists, as well as through web-based inquiries in pain-related social media support groups. Patients who endorsed pain were regarded as eligible for the survey. To be included in the data analysis, fulfilling the diagnosis criteria of chronic pain (International Association for the Study of Pain and International Classification of Diseases, Eleventh Revision, criteria) [2,43] was mandatory. Additional criteria for patients wishing to take part in the study were age ≥18 years, sufficient German-language knowledge, internet access, and providing electronic informed consent. The study was anonymous and voluntary. The survey was designed to be completed in 15 to 20 minutes, and the average time to completion was approximately 20 (SD 7.07) minutes. Of the 525 participants who started the survey and provided informed consent, 342 (65.1%) completed the survey, resulting in a completion rate of 65.1%. Of these 342 participants, 2 (0.6%) reported a *pain duration of < 3 months* and were excluded for not meeting the aforementioned definition of chronic pain. We also excluded the fastest 5% (16/342) and slowest 5% (17/342) of the participants from the study to ensure data quality because slow or fast completion can be an indication of lack of attention and care [44,45]. By excluding extremely fast and slow responders, it was possible to ensure that the data analysis was based on an average sample. This minimized possible biases in response behavior. Thus, of the 525 participants who started the survey and provided informed consent, 307 (58.5%) were included in the data analysis.

### Ethics Approval

The study was conducted in accordance with the Declaration of Helsinki and approved by the ethics committee of the medical faculty of the University of Duisburg-Essen (19-89-47-BO).

### Assessment Instruments

Patient-related data were collected using self-generated items on sociodemographic characteristics, including gender, age, marital status, educational qualifications, employment, and place of residence. Medical data included chronic pain diagnosis criteria, prior treatment, and more detailed description of the symptoms.

Depressive symptoms were screened with the Patient Health Questionnaire depression scale (PHQ-8) [46], in which 8 items assess depressive symptoms on a 4-point Likert scale (from 0=not at all to 3=nearly every day). Scores above the cutoff of 10 indicate major depressive symptoms. The Cronbach α value for this instrument was .84 in this study, indicating high internal consistency. The level of eHealth literacy was assessed with the German version of the eHealth Literacy Scale (eHEALS) [47]. The scale assumes that reading skills are required to use technical offerings, the so-called literacy level. The eHEALS consists of 8 items to measure the skills in this regard. The items are intended to determine knowledge, comfort, and skills in finding, evaluating, and using eHealth information [47]. Sum scores for the eHEALS range from 8 to 40, with higher scores indicating a higher level of eHealth literacy. The Cronbach α value for this instrument was .90 in this study, indicating excellent internal consistency. Using self-generated items, further information concerning the general use of the internet and eHealth was collected. Participants were asked about their private use of media, such as duration and frequency of private internet use and confidence in dealing with eHealth. Internet anxiety was assessed by 3 items regarding concerns about internet use, with answers ranging from 1=strongly disagree to 5=strongly agree. Values above 5 indicate very high internet anxiety. The Cronbach α value for this instrument was .80 in this study, indicating sufficient internal consistency. Data regarding prior eHealth use were also collected.

The UTAUT was used to determine the factors that influence the acceptance of eHealth pain management interventions in patients with chronic pain. The instrument consists of 12 items. Responses are provided using a 5-point Likert scale ranging from 1=totally disagree to 5=totally agree. The added items, including sociodemographic, psychometric, medical, and eHealth-related variables, operate as direct predictors of acceptance. The individual items can be assigned to the predictors PE, EE, and SI. BI, which was operationalized as acceptance, was measured with 3 additional items. In this study, the values for Cronbach α were .90 for PE, .77 for EE, .82 for SI, and .87 for BI (=acceptance), indicating adequate to high internal consistency. More factors were added, including other sociodemographic, medical, and eHealth-related data, as direct predictors of acceptance to the original UTAUT model. The questionnaire with the exact wording of the items is presented in Multimedia Appendix 1 [16,34,38,40,42,48].

### Statistical Analyses

Data analysis was performed using SPSS software (version 26.0; IBM Corp) and R (version 4.0.3; The R Foundation for Statistical Computing). Sum scores for PHQ-8 and eHEALS and mean scores for self-generated items for internet anxiety and eHealth-related knowledge were computed. Furthermore, the UTAUT model with its four scales (PE, EE, SI, and BI) was calculated, and the acceptance (=BI) scores were divided into categories based on previous research [16,38,42]: low acceptance was indicated by scores between 1 and 2.34, moderate acceptance between 2.35 and 3.67, and high acceptance between 3.68 and 5. As Shapiro-Wilk tests revealed that the data were not normally distributed, Wilcoxon rank-sum tests and an ANOVA were used to compare acceptance among the groups (age, number of treatments, treatment effectiveness, cutoff score for PHQ-8, and experience with eHealth). Bonferroni-adjusted α levels were applied. A median split was used to dichotomize age. Multiple hierarchical regression analysis was applied to examine possible predictors of acceptance. Predictors were included blockwise: (1) sociodemographic data, (2) psychometric and medical data, (3) eHealth-related variables, and (4) UTAUT predictors. The extended model was additionally tested against the restricted UTAUT model that only included the UTAUT predictors (PE, EE, and SI). Homoscedasticity was tested through Breusch-Pagan tests. The level of significance was set at α<.05. Effect sizes are presented according to Cohen [49], with values around 0.2, 0.5, and 0.8 being considered small, medium, and large effects, respectively.

## Results

### Sociodemographic, Medical, and Psychometric Data

The vast majority, that is, 92.5% (284/307), of the participants were women, 7.2% (22/307) were men, and 1 (0.3%) self-identified as nonbinary. The mean age of the participants was 45.96 (SD 10.66) years. The participants ranged in age from 18 to 69 years.

Most (280/307, 91.2%) of the patients had endorsed pain for at least 12 months; for 83.1% (255/307), the pain lasted for >2 years. Pain frequency was most frequently reported as permanent (143/307, 46.6%) and daily (102/307, 33.2%). In total, 17.3% (53/307) of the participants had tried >6 different pain treatments. More than half (183/307, 59.6%) of the participants had already received 3 to 6 different pain treatments, and 23.1% (71/307) had tried <3 different pain treatments. More than half (184/307, 59.9%) considered prior treatment efficient. The reported treatments included surgery, medication, psychotherapy, and alternative healing methods. In the PHQ-8 questionnaire, 73.3% (225/307) of the participants achieved a score above the cutoff of 10, indicating depressive symptoms. For further details, refer to Table 1.

Of the 307 participants, 208 (67.8%) had no experience with eHealth pain management interventions. However, regarding digital media use, only 7.8% (24/307) of the participants reported feeling very insecure or a little uncertain. This represents a high level of confidence in the use of digital media in the sample, with 81.4% (250/307) of the participants feeling secure about using digital media (mean 4.17, SD 1.01). The mean level of internet anxiety in this sample was 1.78 (SD 0.83), whereas values above 5 indicate a very high level of internet anxiety. Thus, internet anxiety was low in the sample. On average, the participants showed a high level of eHealth literacy (mean 30.40, SD 5.34) according to eHEALS [47]. For further details, refer to Table 2.

**Table 1:** Sociodemographic, medical, and psychometric data (N=307).

Variable		Value
**Gender, n (%)**		
	Woman	284 (92.5)
	Man	22 (7.2)
	Nonbinary	1 (0.3)
Age (years), mean (SD)		45.96 (10.66)
**Education, n (%)**		
	University and qualification for university	146 (47.6)
	Lower qualification	161 (52.4)
**Occupational status, n (%)**		
	Retired	41 (13.4)
	Employed	176 (57.3)
	Unemployed	51 (16.6)
	Other	39 (12.7)
**Place of residence (population size), n (%)**		
	Large city (>100,000)	86 (28)
	Medium-sized city (>20,000)	91 (29.6)
	Small town (>5000)	60 (19.5)
	Rural municipality (<5000)	70 (22.8)
**Marital status, n (%)**		
	Single	57 (18.6)
	Married	152 (49.5)
	In a relationship	65 (21.2)
	Divorced or separated	29 (9.4)
	Widowed	4 (1.3)
**Pain period, n (%)**		
	3 to 6 months	12 (3.9)
	6 to 12 months	15 (4.9)
	>12 months	280 (91.2)
	1 to 2 years	25 (8.1)
	>2 years	255 (83.1)
**Pain frequency, n (%)**		
	Permanent	143 (46.6)
	Daily	102 (33.2)
	Several times a week	53 (17.3)
	Once a week	9 (2.9)
**Number of prior treatments, n (%)**		
	<3	71 (23.1)
	3 to 6	183 (59.6)
	>6	53 (17.3)
Considered prior treatment efficient, n (%)		184 (62.4)
PHQ-8^a^ (sum score), mean (SD)		13.20 (5.29)
PHQ-8 score <10, n (%)		82 (26.7)

^a^PHQ-8: Patient Health Questionnaire depression scale.

**Table 2:** eHealth-related data (N=307).

Variable				Value
No eHealth experience, n (%)				208 (67.8)
Duration of daily private internet use (hours), mean (SD)				2.9 (1.22)
**Duration of daily private internet use (hours), n (%)**				
	0 to 1		39 (12.7)	
	1 to 2		83 (27)	
	2 to 3		99 (32.2)	
	3 to 4		34 (13.7)	
	>4		44 (14.3)	
Confidence in dealing with eHealth, mean (SD)				4.17 (1.01)
**Confidence in dealing with eHealth, n (%)**				
	Very little confident		10 (3.3)	
	A little unconfident		14 (4.6)	
	Neutral		33 (10.7)	
	Rather confident		106 (34.5)	
	Very confident		144 (46.9)	
Internet anxiety^a^, mean (SD)				1.78 (0.83)
eHealth literacy^b^, mean (SD)				30.40 (5.34)
**UTAUT^c^ core predictors, mean (SD)**				
		Behavioral intention	3.67 (0.89)	
		Social influence	3.46 (0.77)	
		Performance expectancy	3.36 (0.84)	
		Effort expectancy	3.47 (0.79)	

^a^Values above 5 indicate a very high level of internet anxiety (range 1-5).

^b^Higher scores indicate a higher level of eHealth literacy (range 8-40).

^c^UTAUT: Unified Theory of Acceptance and Use of Technology.

### Acceptance of eHealth Pain Management Interventions

General acceptance was moderate to high, with a mean of 3.67 (SD 0.89). In total, 9.1% (28/307) of the participants showed low level of acceptance, 47.2% (145/307) showed moderate level of acceptance, and 43.6% (134/307) showed high level of acceptance.

Of the 307 participants, 154 (50.2%) were below the median age of 47 years, and the mean acceptance score in this group was 3.80 (SD 0.87), whereas 153 (49.8%) had a median age of ≥47 years and had a mean acceptance score of 3.55 (SD 0.89). The Wilcoxon rank-sum test revealed a significantly higher acceptance in the younger age group (*W*=9674.0; *r*=0.156; *P*=.04). Acceptance did not differ significantly among the groups divided by occupational (*F*_3,303_=0.44; *P*=.99) or educational status (*W*=10,515.0; *P*=.76). There were no significant differences in acceptance regarding number of treatments (*F*_2,304_=2.27; *P*=.74), treatment effectiveness (*W*=9037.5; *P*=.67), cutoff for PHQ-8 (*W*=8487.0; *P*=.99), and experience with eHealth (*W*=10,288.0; *P*=.99).

### Predictors of Acceptance of eHealth Pain Management Interventions

Multiple hierarchical regression analysis revealed that the sociodemographic predictors included in the first step explained 4.1% of the variance of acceptance (*R*^²^=0.041; *F*_7,299_=1.83; *P*=.08). In the first step, *age* significantly predicted acceptance (β=–.01; *P*=.004). With the second step, which included the psychometric and medical predictors (*R*^²^=0.069; *F*_13,293_=1.66; *P*=.07), the explained variance increased significantly to 6.9% (*∆R*^²^=0.028; *F*_6,293_=2.97; *P*=.001), although none of the included variables were significant predictors on their own. The eHealth-related predictors included in the third step (*R*^²^=0.130; *F*_25,281_=1.68; *P*=.03) further increased the explained variance significantly to 13% (*∆R*^²^=0.061; *F*_12,281_=4.22; *P*<.001). In the third step, *place of residence: medium-sized city* (β=–.27; *P*=.048), *confidence in dealing with eHealth: neutral* (β=.69; *P*=.04), *confidence in dealing with eHealth: rather confident* (β=.66; *P*=.03), and *confidence in dealing with eHealth: very confident* (β=.71; *P*=.02) were additional significant predictors. The last step included the UTAUT predictors (*R*^²^=0.664; *F*_28,278_=19.63; *P*<.001) and explained 66.4% of the variance in acceptance. The additional predictors increased the explained variance significantly by 53.4% (*∆R*^²^=0.534; *F*_3,278_=147.46; *P*<.001). As expected, *EE* (β=.37), *PE* (β=.33), and *SI* (β=.34) significantly predicted acceptance (all *P*<.001)**.** In addition to the UTAUT predictors, the following variables were found to be significant predictors of acceptance in the overall model (step 4): *place of residence: small town* (β=–.28; *P*=.004), *private daily internet use: 2 to 3 hours* (β=–.25; *P*=.02), and *private daily internet use: >4 hours* (β=–.29; *P*=.02). Table 3 presents an overview of the parameters included in each step in the hierarchical regression model.

**Table 3:** Hierarchical regression model of acceptance (the extended Unified Theory of Acceptance and Use of Technology model; N=307).

Predictor								β^a^						β^b^						T			*R* ^²c^				Δ*R*^²d^			*P* value						
**Step 1: sociodemographic variables**																								0.041				0.041			—^e^					
		Age (years)						–.01						–.11						–2.75			—				—			.006						
		**Occupational status**																																		
							Employed	–.11						–.13						–1.10			—				—			.27						
							Unemployed	–.14						–.15						–1.15			—				—			.25						
							Other	.00						.00						0.09			—				—			.98						
		**Place of residence**																																		
					Medium-sized city					–.26						–.30			–3.12			—				—				.002						
					Small town			–.28					–.31						–2.91			—				—				.004						
					Rural municipality			–.12					–.13						–1.35			—				—				.18						
**Step 2^f^: psychometric and medical variables**																								0.069				0.028			.001					
		PHQ-8^g^						.01						.04						1.08			—				—			.28						
		**Pain duration (months)**																																		
						6 to 12					–.02						–.03		–0.11			—				—				.92						
						12 to 24					–.37						–.41		–1.81			—				—				.07						
						>24					–.18						–.20		–1.04			—				—				.30						
		**Number of treatments tried**																																		
						>6			.05						.06				0.58		—				—				.57							
						<3		–.13					–.15						–1.64			—				—				.10						
**Step 3^f^: eHealth-related factors**																								0.130				0.061			<.001					
	**Private daily internet use (hours)**																																			
				1 to 2						–.17						–.19			–1.51			—				—				.13						
				2 to 3				–.25					–.28						–2.27			—				—				.02						
				3 to 4				–.17					–.19						–1.33			—				—				.19						
				>4				–.29					–.33						–2.27			—				—				.02						
	**Confidence in dealing with eHealth**																																			
				A little unconfident								–.23						–.26		–0.96			—				—			.34						
				Neutral								.28						.31		1.35			—				—			.18						
				Rather confident								.20						.23		1.05			—				—			.29						
				Very confident								.08						.09		0.42			—				—			.68						
				Internet anxiety								–.03						–.03		–0.66			—				—			.51						
				eHealth knowledge								.03						.03		0.83			—				—			.41						
				No eHealth experience								.07						.08		0.92			—				—			.36						
				eHEALS^h^								.00						.02		0.39			—				—			.70						
**Step 4^f^: UTAUT^i^ core predictors**																								0.664				0.534			<.001					
	Effort expectancy									.37						.33			6.74			—				—				<.001						
	Performance expectancy							.33						.31						6.30			—				—			<.001						
	Social influence							.34						.29						6.46			—				—			<.001						

^a^Standardized coefficient beta.

^b^Unstandardized coefficient beta.

^c^Determination coefficient.

^d^Changes in *R*^²^.

^e^Not available.

^f^In steps 2, 3, and 4, only the newly included variables are presented.

^g^PHQ-8: Patient Health Questionnaire depression scale.

^h^eHEALS: eHealth literacy scale.

^i^UTAUT: Unified Theory of Acceptance and Use of Technology.

### Restricted UTAUT Versus Extended UTAUT Model

In our study, the explained variance for the restricted UTAUT with the three core predictors PE, EE, and SI was 61.2% (*R*^²^=0.612, *R*^²^*_adj_*=0.608). The explained variance for our extended UTAUT model with added sociodemographic, medical, and eHealth-related factors reached 66.4% (*R*^²^=0.664, *R*^²^*_adj_*=0.630) of explained variance. The comparison of the 2 models revealed a significant difference in explained variance (*F*_25,278_=1.74; *P*=.02). Thus, the extended UTAUT model explains more variance in the acceptance of eHealth pain management interventions among patients with chronic pain.

## Discussion

### Principal Findings

The main objective of the study was to determine the acceptance of eHealth pain management interventions among patients with chronic pain, as well as identify the factors that influence acceptance. Overall, the acceptance among patients with chronic pain in this study was moderate to high. In total, 43.6% (134/307) of the participants showed a high level of acceptance, 47.2% (145/307) showed a moderate level of acceptance, and only 9.1% (28/307) showed a low level of acceptance.

We were able to confirm the positive relationship between the three core predictors (PE, EE, and SI) of the UTAUT model and acceptance, as demonstrated previously; for example, in patients with diabetes or obesity [34,38,42]. The 3 core predictors of the restricted UTAUT explained 61.2% of the variance in this study. This result is comparable to that in the original study in which the UTAUT was evaluated [50]. This underlines the viability of the UTAUT to be used for eHealth pain management interventions among patients with chronic pain. A comparison of the restricted UTAUT model and the extended UTAUT model revealed a higher explained variance for the extended UTAUT that included the added predictors. The additional factors were included in the extended UTAUT model because additional factors beyond the core predictors can be assumed to influence acceptance.

Age was a significant predictor of acceptance in this study. Young age, defined here as age <47 years, the median age in our sample, was associated with greater acceptance. This is consistent with the results from previous studies [16,39,42]. To increase acceptance among older patients too, they can be especially reached out to when addressing the target group for an intervention. With regard to the influence of SI on acceptance, this could be achieved, for example, through a recommendation by the family physician. We did not include gender in the analysis because our sample was not representative in terms of gender distribution. The place of residence was found to have an influence on acceptance. Patients living in a medium-sized city or small town showed an increased level of acceptance. It is possible that there are fewer face-to-face treatments available in small towns than in large cities. However, when it comes to rural municipalities, we did not observe an increased level of acceptance. This might be comparable to the finding that in rural areas internet-based media platforms are used less often than in cities [51], which might indicate a more reserved attitude and makes individuals from rural areas a relevant target group for interventions that aim to increase acceptance. Education was not significantly associated with acceptance in our study, in contrast to previous studies [16,39]. However, it should be mentioned that an above-average number of participants in the survey have a university degree, and this could at least have had an influence on the high eHealth literacy level in the survey. The lack of effect of education could simply be because people with low education and who might have rated their acceptance level as low are not adequately represented in the study. Of note, this overrepresentation is a common bias in psychological research: 96% of psychological studies are conducted on Western, educated, industrialized, rich, and democratic samples [52,53]. Occupational status was also not significantly associated with acceptance. We did not observe an increased level of acceptance in patients with a higher number of treatment attempts and lower treatment effectiveness. On the one hand, it can be assumed that patients who have already tried various treatments and have still not found an effective treatment may be more willing to try other options, such as web-based interventions. On the other hand, it is possible that patients for whom no treatment has been successful even after many attempts may be suspicious of new forms of treatment because the absence of treatment success has lowered their trust level and perceived self-efficacy. It is possible that these effects may cancel each other out. Internet anxiety was low in the sample, which is in line with overall frequent private internet use and the fact that a proportion of the participants were recruited through the internet. The high level of confidence in the use of digital media underlines the patients’ confidence in using eHealth technologies. Internet anxiety did not have a significant influence on acceptance. However, confidence in dealing with eHealth had an influence on acceptance. Acceptance in participants who felt confident was significantly higher than in those who described their confidence level as neutral. This might provide an opportunity to increase acceptance in the future; for example, the confidence could be increased in advance through training or tutorials by providing comprehensive information and personal assistance during the use of eHealth pain management interventions. Furthermore, it is noteworthy that despite frequent private internet use, only 32.2% (99/307) of the participants had already had experience with eHealth. It is unclear whether this is due to a shortage of eHealth offerings in everyday clinical practice or previous skepticism about eHealth. Nevertheless, prior experience with eHealth had no influence on acceptance in this study.

In contrast to previous studies [42], we observed no association between depressive symptoms and acceptance of eHealth pain management interventions. The reasons for the high proportion (225/307, 73.3%) of participants with depressive symptoms may be multifactorial. First, the proportion of patients with depressive symptoms is higher among patients with chronic pain than in the general population [54-58]. Second, the data collection took place during the COVID-19 pandemic when the prevalence of depression was elevated [59]. However, the fact that an excessively high proportion (225/307, 73.3%) of participants in our sample showed depressive symptoms underlines the comorbidity of chronic pain and mental health burden [54,55]. Interestingly, acceptance decreased with increasing duration of private daily internet use. An explanation for this finding could be that participants who already use the internet a lot in their daily lives do not want to further extend the hours of internet use and therefore do not want to seek therapy through the internet. To assess this more accurately, it would be useful to conduct future studies to determine how patients with chronic pain use the internet and whether internet use itself is associated with acceptance. It would be interesting to know whether they associate internet use with work or see it more as entertainment, which they would like to keep separate from serious topics such as the treatment of their illnesses. In this case, future studies could also include questions on the circumstances in which participants judge offerings as serious.

Although we did not find an influence on the acceptance of eHealth pain management interventions for several of the aforementioned factors, overall acceptance was higher than in previous studies [37,38,40-42]. Several explanations for this can be considered. First, patients were also recruited through social media, and social media users might generally be more accepting of web-based programs. The fact that mainly women participated in the survey should also be noted. In a previous study with patients with diabetes, acceptance of eHealth interventions was significantly higher among women than among men [38]. Understanding the reasons behind possible gender differences in eHealth acceptance would thus be necessary to improve eHealth acceptance among men. An alternative explanation could be that patients with chronic pain generally show higher acceptance than other groups. To further investigate this, patients with chronic pain would need to be compared with other patient populations. Another reason for the higher acceptance could be the timing of data collection. It is likely that acceptance of eHealth interventions changes with their increasing implementation in health care [60]. In addition, younger people are already showing increased acceptance, and as they will make up a large part of the population in the future because of demographic change, increased acceptance can also be expected as a result.

Even with the extended UTAUT, only selected factors were tested for their influence on the acceptance of eHealth pain management interventions. It is likely that there are additional factors that need to be investigated to further understand acceptance and implementation of eHealth pain management interventions. Further research should target these influences.

### Limitations

The results of the study should be interpreted in the context of the limitations discussed herein. A proportion of participants were reached through inquiries in web-based support groups. Part of the reason for this may be the timing of data collection. Because of the COVID-19 pandemic, for example, time spent by patients waiting inside physicians’ offices was reduced, and hand-distributed flyers were accepted reluctantly, which made offline recruitment more difficult. It is quite possible that by recruiting through the internet, mainly those who are already more willing to use the internet were reached. Thus, a selection bias cannot be ruled out. In future surveys, more emphasis should be placed on recruiting through in-person channels. Because of the predominance of female participants, the influence of gender could not be investigated. The excessive proportion (284/307, 92.5%) of women in the sample is not representative of the gender composition in the overall population of patients with chronic pain, which limits the generalizability of the results because of sampling bias. A reason for this could be that recruitment widely took place in social media groups, which largely consist of female members, including groups for people with endometriosis and fibromyalgia, where the majority of those affected are women [61,62]. Furthermore, the study provides only a theoretical account of whether patients with chronic pain would be willing to use eHealth pain management interventions. If one regards acceptance as BI to use such offerings, the extent of their actual use remains to be determined. However, considering the intention-behavior gap [63], that is, the phenomenon that the intention to do something does not lead to real behavior to the same extent, it remains unclear whether the observed intention will also lead to actual use. Future studies should thus compare survey results of acceptance with subsequent use of such interventions. Nevertheless, knowledge of the factors influencing acceptance should be used and specifically addressed in the development of eHealth pain management interventions.

### Conclusions

This study was able to demonstrate overall moderate to high acceptance of eHealth pain management interventions among patients with chronic pain. This high rate of acceptance suggests that eHealth interventions can offer a viable alternative for situations in which face-to-face treatment is not possible. The factors PE, EE, and SI were core predictors of acceptance. The extended UTAUT proved to be a useful tool for determining acceptance as well as the factors that influence the acceptance of eHealth pain management interventions among patients with chronic pain. Understanding the factors that influence acceptance is important to provide tailored eHealth pain management interventions and promote their actual use. When access to face-to-face treatment is limited, eHealth interventions offer a good alternative. With all that, we emphasize that the aim is not to replace face-to-face treatment but to complement it; for example, eHealth interventions can help bridge the gap until face-to-face therapy is received or complement existing therapies. Finally, this study highlights the importance of taking patients’ expectations, needs, and capabilities into account when developing new treatment approaches.

## Appendix

Multimedia Appendix 1 The used Unified Theory of Acceptance and Use of Technology.

## Abbreviations

BI: behavioral intention
EE: effort expectancy
eHEALS: eHealth literacy scale
FC: facilitating condition
PE: performance expectancy
PHQ-8: Patient Health Questionnaire depression scale
SI: social influence
UTAUT: Unified Theory of Acceptance and Use of Technology

## References

[1] Raja SN, Carr DB, Cohen M, Finnerup NB, Flor H, Gibson S, Keefe FJ, Mogil JS, Ringkamp M, Sluka KA, Song XJ, Stevens B, Sullivan MD, Tutelman PR, Ushida T, Vader K (2020). The revised International Association for the Study of Pain definition of pain: concepts, challenges, and compromises. Pain.

[2] Treede RD, Rief W, Barke A, Aziz Q, Bennett MI, Benoliel R, Cohen M, Evers S, Finnerup NB, First MB, Giamberardino MA, Kaasa S, Kosek E, Lavand'homme P, Nicholas M, Perrot S, Scholz J, Schug S, Smith BH, Svensson P, Vlaeyen JW, Wang SJ (2015). A classification of chronic pain for ICD-11. Pain.

[3] Häuser W, Schmutzer G, Hilbert A, Brähler E, Henningsen P (2015). Prevalence of chronic disabling noncancer pain and associated demographic and medical variables: a cross-sectional survey in the general German population. Clin J Pain.

[4] Koesling D, Kieselbach K, Bozzaro C (2019). Chronischer Schmerz und Gesellschaft : Soziologische Analyse einer komplexen Verschränkung. Schmerz.

[5] Rice AS, Smith BH, Blyth FM (2016). Pain and the global burden of disease. Pain.

[6] Sessle B (2011). Unrelieved pain: a crisis. Pain Res Manag.

[7] Cáceres-Matos R, Gil-García E, Barrientos-Trigo S, Porcel-Gálvez AM, Cabrera-León A (2020). Consequences of chronic non-cancer pain in adulthood. Scoping review. Rev Saude Publica.

[8] Andrew R, Derry S, Taylor RS, Straube S, Phillips CJ (2014). The costs and consequences of adequately managed chronic non-cancer pain and chronic neuropathic pain. Pain Pract.

[9] Phillips CJ (2009). The cost and burden of chronic pain. Rev Pain.

[10] Wolff R, Clar C, Lerch C, Kleijnen J (2011). Epidemiologie von nicht tumorbedingten chronischen Schmerzen in Deutschland. Schmerz.

[11] Nadstawek J, Bachmann J, Straßmeir W (2019). BVSD Weissbuch Schmerzmedizin 2019. Berufsverband der Ärzte und Psychologischen Psychotherapeuten in der Schmerz- und Palliativmedizin in Deutschland.

[12] Lynch ME, Campbell F, Clark AJ, Dunbar MJ, Goldstein D, Peng P, Stinson J, Tupper H (2008). A systematic review of the effect of waiting for treatment for chronic pain. Pain.

[13] Oh H, Rizo C, Enkin M, Jadad A (2005). What is eHealth (3): a systematic review of published definitions. J Med Internet Res.

[14] Bakitas M, Cheville AL, Mulvey TM, Peppercorn J, Watts K, Dionne-Odom JN (2021). Telehealth strategies to support patients and families across the cancer trajectory. Am Soc Clin Oncol Educ Book.

[15] Kitamura C, Zurawel-Balaura L, Wong RK (2010). How effective is video consultation in clinical oncology? A systematic review. Curr Oncol.

[16] Hennemann S, Beutel ME, Zwerenz R (2016). Drivers and barriers to acceptance of Web-based aftercare of patients in inpatient routine care: a cross-sectional survey. J Med Internet Res.

[17] Meier CA, Fitzgerald MC, Smith JM (2013). eHealth: extending, enhancing, and evolving health care. Annu Rev Biomed Eng.

[18] Kluge EH (2011). e-Health promises and challenges: some ethical considerations. Stud Health Technol Inform.

[19] Hedman E, Andersson E, Ljótsson B, Andersson G, Rück C, Lindefors N (2011). Cost-effectiveness of Internet-based cognitive behavior therapy vs. cognitive behavioral group therapy for social anxiety disorder: results from a randomized controlled trial. Behav Res Ther.

[20] Macea DD, Gajos K, Daglia Calil YA, Fregni F (2010). The efficacy of Web-based cognitive behavioral interventions for chronic pain: a systematic review and meta-analysis. J Pain.

[21] Eccleston C, Blyth FM, Dear BF, Fisher EA, Keefe FJ, Lynch ME, Palermo TM, Reid MC, Williams AC (2020). Managing patients with chronic pain during the COVID-19 outbreak: considerations for the rapid introduction of remotely supported (eHealth) pain management services. Pain.

[22] Barak A, Hen L, Boniel-Nissim M, Shapira N (2008). A comprehensive review and a meta-analysis of the effectiveness of Internet-based psychotherapeutic interventions. J Technol Human Serv.

[23] Carlbring P, Andersson G, Cuijpers P, Riper H, Hedman-Lagerlöf E (2018). Internet-based vs. face-to-face cognitive behavior therapy for psychiatric and somatic disorders: an updated systematic review and meta-analysis. Cogn Behav Ther.

[24] Eccleston C, Fisher E, Craig L, Duggan GB, Rosser BA, Keogh E (2014). Psychological therapies (Internet-delivered) for the management of chronic pain in adults. Cochrane Database Syst Rev.

[25] Schneider LH, Hadjistavropoulos HD (2014). When in doubt, ask the audience: potential users' perceptions of Internet-delivered cognitive behavioural therapy for chronic pain. Pain Res Manag.

[26] Ruehlman LS, Karoly P, Enders C (2012). A randomized controlled evaluation of an online chronic pain self management program. Pain.

[27] Linardon J, Cuijpers P, Carlbring P, Messer M, Fuller-Tyszkiewicz M (2019). The efficacy of app-supported smartphone interventions for mental health problems: a meta-analysis of randomized controlled trials. World Psychiatry.

[28] Andersson G (2016). Internet-delivered psychological treatments. Annu Rev Clin Psychol.

[29] Cranen K, Drossaert CH, Brinkman ES, Braakman-Jansen AL, Ijzerman MJ, Vollenbroek-Hutten MM (2012). An exploration of chronic pain patients' perceptions of home telerehabilitation services. Health Expect.

[30] Rosser BA, Eccleston C (2011). Smartphone applications for pain management. J Telemed Telecare.

[31] Portelli P, Eldred C (2016). A quality review of smartphone applications for the management of pain. Br J Pain.

[32] Lalloo C, Jibb LA, Rivera J, Agarwal A, Stinson JN (2015). "There's a pain app for that": review of patient-targeted smartphone applications for pain management. Clin J Pain.

[33] Sundararaman LV, Edwards RR, Ross EL, Jamison RN (2017). Integration of mobile health technology in the treatment of chronic pain: a critical review. Reg Anesth Pain Med.

[34] Venkatesh V, Morris MG, Davis GB, Davis FD (2003). User acceptance of information technology: toward a unified view. MIS Q.

[35] Harst L, Lantzsch H, Scheibe M (2019). Theories predicting end-user acceptance of telemedicine use: systematic review. J Med Internet Res.

[36] Kaltenthaler E, Sutcliffe P, Parry G, Beverley C, Rees A, Ferriter M (2008). The acceptability to patients of computerized cognitive behaviour therapy for depression: a systematic review. Psychol Med.

[37] Philippi P, Baumeister H, Apolinário-Hagen J, Ebert DD, Hennemann S, Kott L, Lin J, Messner EM, Terhorst Y (2021). Acceptance towards digital health interventions - Model validation and further development of the Unified Theory of Acceptance and Use of Technology. Internet Interv.

[38] Damerau M, Teufel M, Musche V, Dinse H, Schweda A, Beckord J, Steinbach J, Schmidt K, Skoda E, Bäuerle A (2021). Determining acceptance of e-mental health interventions in digital psychodiabetology using a quantitative Web-based survey: cross-sectional study. JMIR Form Res.

[39] Crisp DA, Griffiths KM (2014). Participating in online mental health interventions: who is most likely to sign up and why?. Depress Res Treat.

[40] Baumeister H, Seifferth H, Lin J, Nowoczin L, Lüking M, Ebert D (2015). Impact of an acceptance facilitating intervention on patients' acceptance of Internet-based pain interventions: a randomized controlled trial. Clin J Pain.

[41] Lin J, Faust B, Ebert DD, Krämer L, Baumeister H (2018). A Web-based acceptance-facilitating intervention for identifying patients' acceptance, uptake, and adherence of internet- and mobile-based pain interventions: randomized controlled trial. J Med Internet Res.

[42] Rentrop V, Damerau M, Schweda A, Steinbach J, Schüren LC, Niedergethmann M, Skoda EM, Teufel M, Bäuerle A (2022). Predicting acceptance of e-mental health interventions in patients with obesity by using an extended unified theory of acceptance model: cross-sectional study. JMIR Form Res.

[43] (2021). ICD-11 for Mortality and Morbidity Statistics (ICD-11 MMS). World Health Organization.

[44] Greszki R, Meyer M, Schoen H (2015). Exploring the effects of removing "too fast" responses and respondents from Web surveys. Public Opin Q.

[45] Kato T, Miura T (2021). The impact of questionnaire length on the accuracy rate of online surveys. J Market Anal.

[46] Kroenke K, Strine TW, Spitzer RL, Williams JB, Berry JT, Mokdad AH (2009). The PHQ-8 as a measure of current depression in the general population. J Affect Disord.

[47] Norman CD, Skinner HA (2006). eHEALS: the eHealth literacy scale. J Med Internet Res.

[48] Ebert DD, Berking M, Cuijpers P, Lehr D, Pörtner M, Baumeister H (2015). Increasing the acceptance of Internet-based mental health interventions in primary care patients with depressive symptoms. A randomized controlled trial. J Affect Disord.

[49] Cohen J (1988). Statistical Power Analysis for the Behavioral Sciences.

[50] Otto L, Harst L, Schlieter H, Wollschlaeger B, Richter P, Timpel P (2018). Towards a unified understanding of eHealth and related terms – proposal of a consolidated terminological basis. Proceedings of the 11th International Joint Conference on Biomedical Engineering Systems and Technologies - (Volume 5).

[51] Perrin A (2015). Social media usage: 2005-2015. Pew Research Center.

[52] Arnett JJ (2008). The neglected 95%: why American psychology needs to become less American. Am Psychol.

[53] Darling N (2017). Attracting WEIRD Samples. Pschology Today.

[54] Wong WS, Chen PP, Yap J, Mak KH, Tam BK, Fielding R (2011). Chronic pain and psychiatric morbidity: a comparison between patients attending specialist orthopedics clinic and multidisciplinary pain clinic. Pain Med.

[55] Breivik H, Collett B, Ventafridda V, Cohen R, Gallacher D (2006). Survey of chronic pain in Europe: prevalence, impact on daily life, and treatment. Eur J Pain.

[56] Surah A, Baranidharan G, Morley S (2014). Chronic pain and depression. Continuing Educ Anaesthesia Crit Care Pain.

[57] Magni G, Marchetti M, Moreschi C, Merskey H, Luchini SR (1993). Chronic musculoskeletal pain and depressive symptoms in the National Health and Nutrition Examination. I. Epidemiologic follow-up study. Pain.

[58] Bair MJ, Robinson RL, Katon W, Kroenke K (2003). Depression and pain comorbidity: a literature review. Arch Intern Med.

[59] Lakhan R, Agrawal A, Sharma M (2020). Prevalence of depression, anxiety, and stress during COVID-19 pandemic. J Neurosci Rural Pract.

[60] Baumeister H, Lin J, Ebert DD (2017). [Internet- and mobile-based approaches : psycho-social diagnostics and treatment in medical rehabilitation]. Bundesgesundheitsblatt Gesundheitsforschung Gesundheitsschutz.

[61] Neumann L, Buskila D (2003). Epidemiology of fibromyalgia. Curr Pain Headache Rep.

[62] Parasar P, Ozcan P, Terry KL (2017). Endometriosis: epidemiology, diagnosis and clinical management. Curr Obstet Gynecol Rep.

[63] Sheeran P, Webb TL (2016). The intention-behavior gap. Soc Pers Psychol Compass.

