# Supplementary material for: Determining the Influencing Factors on Acceptance of eHealth Pain Management Interventions Among Patients With Chronic Pain Using the Unified Theory of Acceptance and Use of Technology: Cross-sectional Study
Source: JMIR Form Res. 2022 Aug 17;6(8):e37682. doi: 10.2196/37682 (PMC9434396; doi:10.2196/37682)
Supplement: Multimedia Appendix 1 [file formative_v6i8e37682_app1.docx]

Appendix 1. The used Unified Theory of Acceptance and Use of Technology.

| **Outcome** | **Items** |
| --- | --- |
| Behavioral Intention/ Acceptance | "I would like to try *an online pain management intervention*. " [40, 48]  "I would use *an online pain management intervention* if offered to me." [40, 48]  "I would recommend *an online pain management intervention* to my friends." [40, 48] |
| Social Influence (SI) | "People close to me would approve of the use of *an online pain management intervention*." [34, 40, 48]  "My primary care physician would approve the use of *an online pain management intervention*." [40, 48]  "My friends would approve of *an online pain management intervention*." [16] |
| Performance Expectancy (PE) | "*An online pain management intervention* could improve my general well-being." [40, 48]  "*An online pain management intervention* could help me with stress." [40, 48]  "*An online pain management intervention* could help me improve my personal (psychological) health." [40, 48] |
| Effort Expectancy (EE) | "The use of *an online pain management intervention* would not be an additional burden for me." [38, 42].  "*An online pain management intervention* would be easy to operate and comprehend." [34, 40, 48]  "I could arrange using a*n online pain management intervention* in my everyday life." [15, 40, 48] |

*Note.* Italicized verbalizations have been adapted.
